# Limits to Crystallization Pressure

**DOI:** 10.1021/acs.langmuir.2c01325

**Published:** 2022-09-09

**Authors:** Lei Li, Felix Kohler, Joanna Dziadkowiec, Anja Røyne, Rosa M. Espinosa Marzal, Fernando Bresme, Espen Jettestuen, Dag Kristian Dysthe

**Affiliations:** † Physics of Geological Processes (PGP), The NJORD Centre, Department of Physics, University of Oslo, PO box 1048 Blindern, 0316 Oslo, Norway; ‡ Environmental Engineering and Science, Department of Civil and Environmental Engineering, University of Illinois at Urbana−Champaign, Urbana, Illinois 61801, United States; § Department of Chemistry, Molecular Sciences Research Hub, Imperial College, W12 0BZ, London, United Kingdom; ∥ Norce Research, Essendropsgate 3, 0368 Oslo, Norway

## Abstract

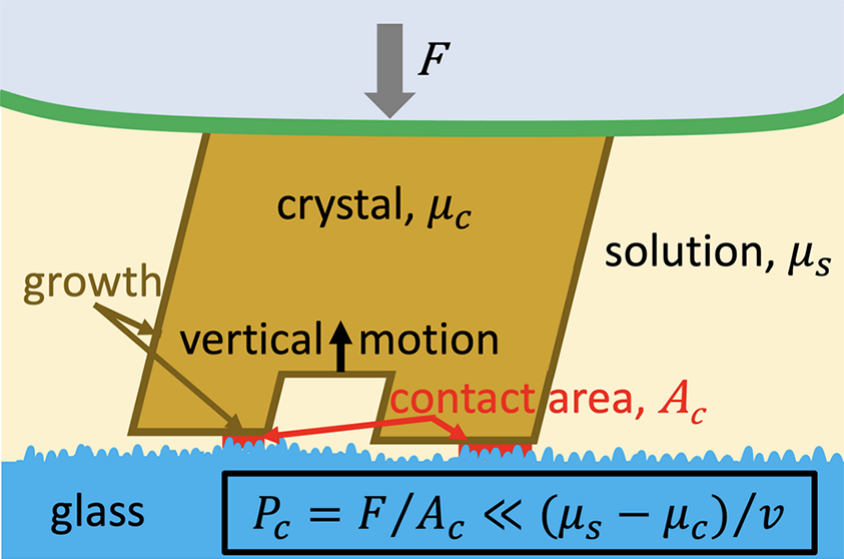

Crystallization pressure drives deformation and damage in monuments, buildings, and the Earth’s crust. Even though the phenomenon has been known for 170 years, there is no agreement between theoretical calculations of the maximum attainable pressure and experimentally measured pressures. We have therefore developed a novel experimental technique to image the nanoconfined crystallization process while controlling the pressure and applied it to calcite. The results show that displacement by crystallization pressure is arrested at pressures well below the thermodynamic limit. We use existing molecular dynamics simulations and atomic force microscopy data to construct a robust model of the disjoining pressure in this system and thereby calculate the absolute distance between the surfaces. On the basis of the high-resolution experiments and modeling, we formulate a novel mechanism for the transition between damage and adhesion by crystallization that may find application in Earth and materials sciences and in conservation of cultural heritage.

## Introduction

Crystallization pressure is well-known to induce fracture and deformation in solids confining crystals, damaging buildings and monuments,^1,2^ lifting layers of the Earth’s surface^3^ and it is thought to drive vein formation,^4,5^ spheroidal weathering^6^ and cracking during metamorphism and frictional failure of the Earth’s crust.^7^

The question of what limits the crystallization pressure is important to mitigation, repair and conservation of buildings and monuments damaged by “salt crystallization”. Different treatments in stone conservation aim at altering the surface energy of pore surfaces in order to limit water transport, controlling the regions where crystallization occurs and by limiting the crystallization pressure itself.^1,8−10^ In Earth sciences, it is fundamentally important to know if a weathering reaction or a metamorphic reaction may generate a crystallization pressure sufficient to fracture the surrounding rock, opening new fluid pathways for further reaction and frictional failure. Recent estimates for olivine hydration and carbonation suggest pressures of the order of 1 GPa can be reached,^7^ whereas recent experiments show that the fracture process driven by crystallization pressure closes down long before such a pressure limit is reached.^11^

The crystallization pressure is generated by a crystal growing in a “load bearing” grain boundary/contact area. A load bearing grain boundary/contact area is an area where the solid grains transmit stress to each other (1) either through direct interatomic interactions between the solids, a solid–solid contact, (2) or transmitted through a thin layer of fluid, where the pressure supported by the fluid is called the disjoining pressure. In the first case, the solid nature of the grain boundary inhibits mass transport except at high temperature. In the second case, mass may be transported in the fluid layer to the growing crystal. The driving force of the mass transport, crystal growth and crystallization pressure is the supersaturation of the fluid present. The existence of a crystallization pressure has been observed and demonstrated many times during the last 170 years^4,9,12−20^ and the thermodynamic limit to this pressure, *P*_*c*_ has been known since the work of Correns and Steinborn:^14,15^*P*_c_ = Δμ_s_/*v*, where Δμ_s_ is chemical potential of the solute in solution relative to the equilibrium state and *v* is the molar volume of the crystal. Apart from the somewhat dubious results of Correns,^14,15^ no-one has ever reported crystallization pressures approaching the thermodynamic limit.^9,15−17^ There are three main candidates to explain the discrepancy: (1) As already observed in 1915, the load bearing contact area is much smaller than the apparent contact area,^13,17,19−21^ meaning that the pressure in the load bearing contact area may possibly be approaching the thermodynamic limit. (2) Due to mass transport by diffusion, the supersaturation in the contact is smaller than in the bulk solution.^16,18,19,21^ (3) The fluid film in the contact “collapses”, a stable, “close contact” is created and diffusion mass transport and crystal growth stops before the thermodynamic limit of crystallization pressure is reached.^21^

In order to understand what limits the disjoining pressure, we develop a new experimental setup where we control and measure the exact supersaturation, the real load bearing contact area, the crystal growth rate, and we use existing simulation results and AFM data to construct a model of the disjoining pressure and diffusion of the system that we study experimentally. We conclude that the equilibrium concepts of crystallization pressure and disjoining pressure are not sufficient to explain the experimental results. Hence, we propose a new mechanism: The reactive surface grows locally to increase the adhesive surface area and the adhesive energy between the solid surfaces separated by 3–4 water layers becomes larger than the energy associated with the supersaturation driving the crystallization (see Figure 6).

## Experimental Section

The experiments presented here are performed at room temperature and have been designed for *in situ* observations of nanoconfined calcite growth under highly controlled conditions. The microfluidic setup provides a very accurate and stable supersaturation and has a high degree of control of the pressure at the confined surface. The topography of the nanoconfined calcite surface and thereby the load bearing contacts (glass-calcite distance *h* < 20 nm) are recorded with nanometer vertical resolution during the whole growth process by high resolution reflection interference contrast microscopy (RICM).

We have previously established how to accurately control supersaturation during growth of calcite in a microfluidic device.^20^ We have also previously shown that we can measure the thickness of the fluid film that excerts a disjoining pressure between a calcite crystal and the supporting glass surface and that this agrees well with DLVO theory.^22^ When the calcite grows in the load bearing calcite–glass contact it “pushes itself away” from the glass and excerts a crystallization pressure equal to the disjoining pressure in the fluid film of the load bearing contact. We have previously shown that we can accurately quantify this growth rate.^22^ The novelty of the experiment described here with respect to previous versions^20,22^ is a microfluidic control of the force on the growing crystal. This allows us to probe how the growth rate depends on the disjoining pressure. The basic idea of the experiment is this: If the growth rate goes to zero the maximum crystallization pressure has been reached (or passed).

### Microfluidic Device with Pressure Control Channel

**Figure 1:**
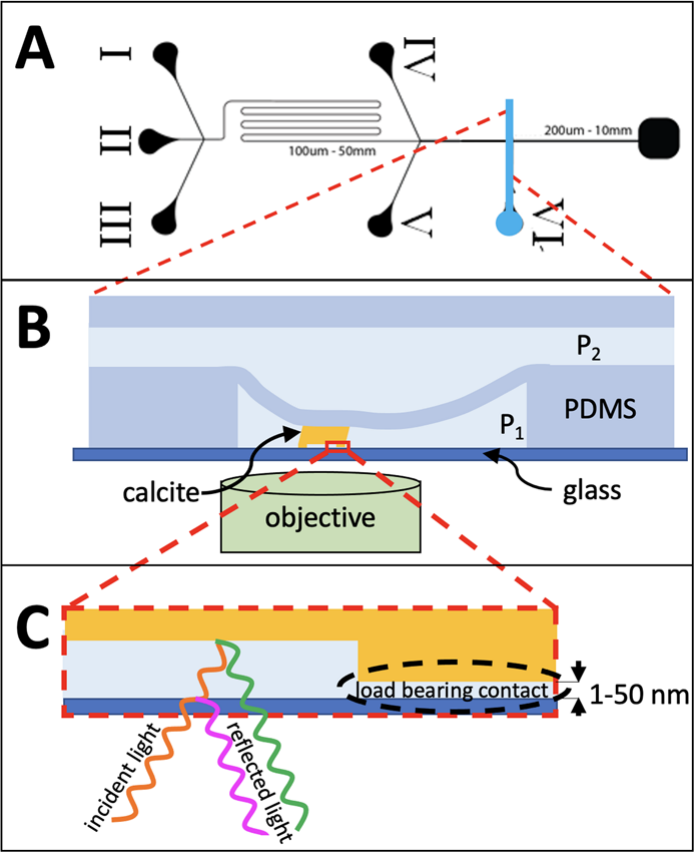
Microfluidic experiment with growth control, pressure control, and in situ interferometric (RICM) imaging. (A) Flow control pattern with five inlets I–V in the lower (flow) layer to vary the CaCO_3_ concentration depending on the relative flow rates: (I) 2 mM Na_2_CO_3_, (II) water, (III) 2 mM CaCl_2_, (IV) 10 mM Na_2_CO_3_, and (V) 10 mM CaCl_2_. Inlets IV and V are used for nucleation only and are closed during growth. Inlet VI (blue) controls pressure *P*_2_ in the upper layer. (B) Two layers of fluid control separated by a PDMS membrane. Lower layer: flow and concentration control (pressure *P*_1_) via inlets I–V (as published in ref (20)) and, novel in this study, upper layer for control of force between calcite and glass by *P*_2_ > *P*_1_ controlled via inlet VI. (C) In situ imaging with height measurement by interference between light reflected from glass-fluid interface (pink) and fluid-calcite interface (green). This allows identification of load bearing contacts and crystal growth rate in these contacts.

The microfluidic device, which is shown in Figure 1, consists of a cover glass with two PDMS layers on top. The PDMS is attached to the glass and defines two layers of fluid channels. The lower layer is used to control nucleation and growth of calcite, the upper layer is used to control contact pressure between crystal and glass.

The layout of the lower layer corresponds to the one described previously.^22^ However, it is only 29 ± 0.3 μm deep. The layer has 5 inlets (I–V) with the following fluid concentrations: (I) 2 mM Na_2_CO_3_, (II) water, (III) 2 mM CaCl_2_, (IV) 10 mM Na_2_CO_3_, and (V) 10 mM CaCl_2_. The CaCl_2_, H_2_O and Na_2_CO_3_ solutions mix in the main channel by diffusion and the relative flow rates determine the final CaCO_3_ concentration. To induce nucleation, we use inlets IV and V to produce nuclei that attach to the walls of the channel. Inlets IV and V are used for nucleation only and are closed during growth. Multiple nucleations or nuclei at undesired locations are dissolved by lowering the concentration of the solution. Calcite nucleation and dissolution of nuclei is repeated until a nucleus is attached on the PDMS membrane in the desired region. After nucleation, a CaCO_3_ concentration of 0.801 ± 0.002 mM has been used, which corresponds to a saturation index of Ω = 0.44.^22^

Novel in this study is the use of the upper fluid layer to control the force on the crystal. The fluid pressure in the upper fluid layer can be increased in order to bend the 6 μm thick PDMS membrane between the two fluid layers and push the calcite crystal toward the glass. In this manner, the force between the calcite crystal and the glass can be controlled. Before the calcite crystal comes into contact with the glass bottom of the microfluidic channel the pressure *P*_2_ – *P*_1_ deforms the membrane. Once a load bearing contact is achieved the force on the crystal from the membrane is proportional to the change in pressure *P*_2_ times the area, *A*_m_, of the membrane closest to the crystal, *F*_c_ = Δ*P*_2_*A*_m_ – *k*Δ*z* (see first section Supporting Information), where *k* is the “spring constant” of the membrane and Δ*z* is the vertical displacement of the top surface of the crystal.

### Reflection Interference Contrast Microscopy

#### Fluid Film Thickness *h*(*x*, *y*)

The inverted microscope under the microfluidic device illuminates the crystal through the objective and receives light reflected from the glass–liquid interface and from the crystal–liquid interface (see Figure 1). The two reflections interfere and form an image (*xy*-plane) of the confined crystal interface with local intensity *I*(*x*, *y*) that depends on the fluid film thickness or local distance *h*(*x*, *y*) between the glass surface and the crystal
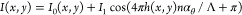
1where *n* = 1.33 is the refractive index of water, Λ = 550 nm is the wavelength of our light source, *I*_0_ is the background intensity, and *I*_1_/*I*_0_ is the contrast and α_θ_ ≈ 1 is a factor that accounts for the effective angle of the light with respect to the optical axis. Because *I*_0_(*x*, *y*) varies across the image due to refractions and nonuniform reflections at other surfaces of the crystal, there is an uncertainty of about ±10 nm in the determination of contact (*h* = 0). The details of reflection interference contrast microscopy (RICM) have been explained in detail elsewhere.^19,20^

#### Load Bearing Contact Area

**Figure 2:**
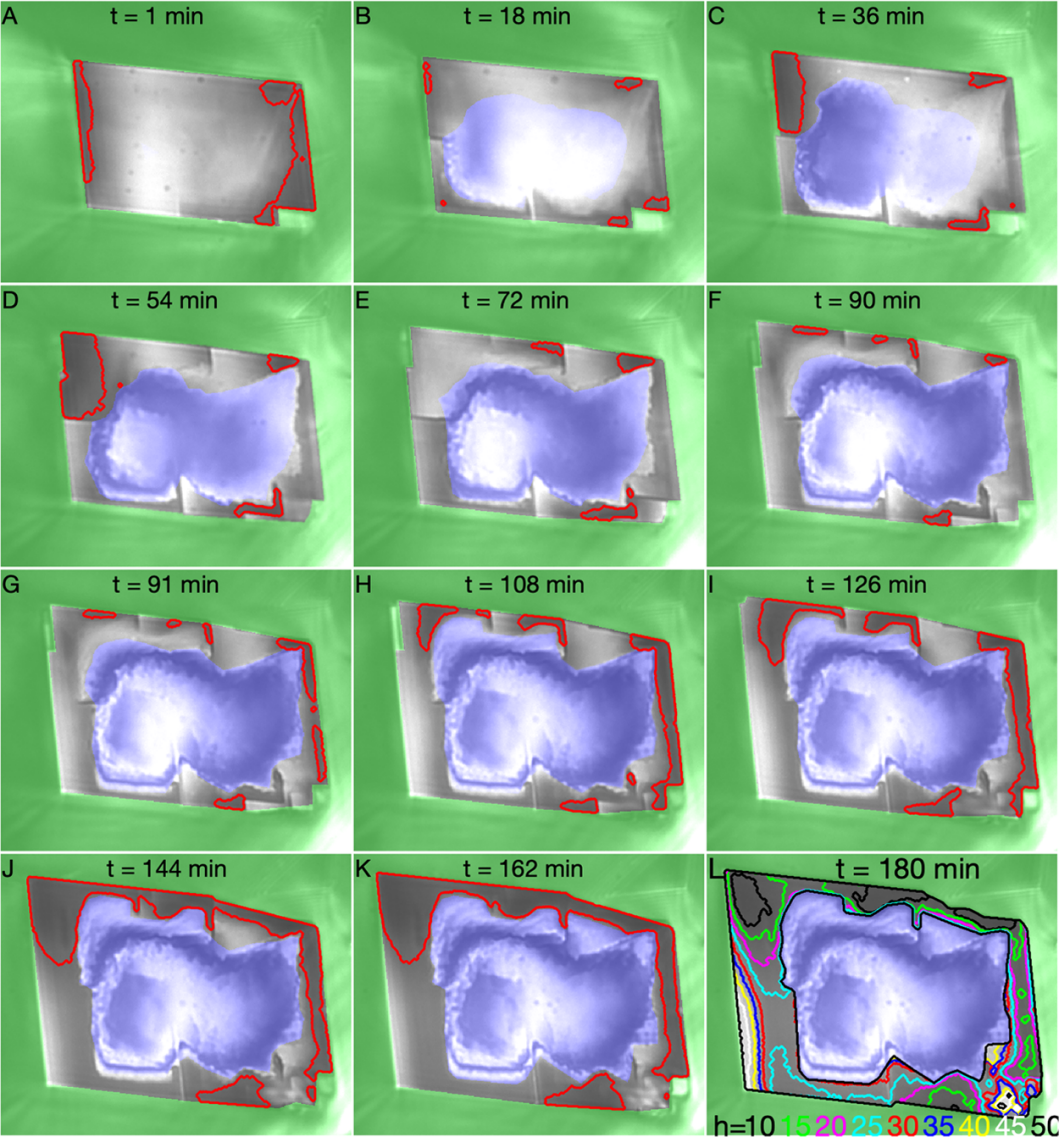
Nanoconfined calcite surface evolution. RICM images of the nanoconfined crystal growth of calcite at low load (top six images) and high load (bottom six images). Each image is 27 × 21 μm. The intensity (brightness), *I*, in the images indicate height, *h*, (fluid film thickness) above the glass surface: *I* ∝ −cos(2*πh*/207 nm). In panel L, the contours of different heights are drawn, in the other images the contour of *h* = 20 nm is drawn. The growth rim of the crystal is in grayscale intensity, the interior cavity with no growth is shown with a blue-white intensity scale and the exterior is tinted green. Images A–F: Evolution from *t* = 0 when saturation index of the solution is increased from 0 to 0.44, applied pressure is *P*_2_ = 20 kPa and the force transmitted to the crystal is *F* ≈ 7 nN. At this low load the load bearing contacts change dynamically. Images G–L: Evolution from *t* = 91 min when applied pressure is increased to *P*_2_ = 30 kPa and the force transmitted to the crystal is *F* = 64 μN. At this high load, the load bearing contacts merge and grow. A timelapse movie of the experiment is available as Supporting Information.

In Figure 2B, the contours of different *h* from 10 to 50 nm as determined by eq 1 are shown. For our calcite–glass system with 0.8 mM CaCO_3_ and NaCl concentrations, the main trend of the disjoining pressure is *P* ∝ exp(*h*/λ_*D*_), where λ_D_ = 7.8 nm is the Debye length calculated according the Supporting Information of Diao and Espinosa-Marzal^24^ and *P*(*h* = 50 nm) ≈ 1 Pa and *P*(*h* = 10 nm) ≈ 100 Pa. The area inside any of the contours in Figure 2 B can thus be defined as a load bearing contact area *A*_c_. Since *A*_c_(*h*) is a monotonous function of *h* and we find that all *A*_c_(*h*, *t*) for *h* ∈ [10, 50] nm have the same features (see second section in Supporting Information) we chose to use *A*_c_(*h* = 20 nm) as the practical definition of load bearing contact area in the following. The outer contours of this load bearing contact area are indicated in Figure 2.

#### Force between Crystal and Glass

The crystal is attached to an elastic membrane that is stretched by increasing the control pressure *P*_2_. The elastic constant *k* = 8 ± 3 N/m is calculated from the displacement of the crystal before it touches the glass. At control pressure *P*_2,0_ = 20 kPa the crystal is gently pressed flat onto the glass. The contact pressure *P*_c,0_ = 20 ± 10 Pa at this reference state is known from earlier experiments,^22^ and the load bearing contact area *A*_c,0_ = 370 μm^2^ is found from the RICM images as explained above. The contact force at this reference state is therefore *F*_c,0_ = *P*_c,0_*A*_c,0_ = 7 ± 3 nN. When the control pressure is increased by Δ*P*_2_ = 10 kPa the contact force is increased to
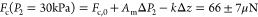
2where *A*_m_ is the representative membrane area and Δ*z* is the vertical height change of the crystal. For further details, see the first section of Supporting Information.

#### Upward Growth Rate

The upward growth rate of the crystal d*z*/d*t*, where *z* is the vertical position of the crystal, and *t* the time, is determined by the interferometric images. Once the crystal reaches a certain size all growth on the confined surface of the crystal will happen at a rim around the outer edge of the surface and inside this rim there will be a region [*x*,*y*]_0_ of no growth.^19^ The growth rim and cavity with no growth are indicated with different colors in Figure 2A–L. The change in height *h*([*x*,*y*]_0_) in the regions [*x*,*y*]_0_ of the crystal surface where there is no growth is thus equal to the change in the vertical crystal position: Δ*z*(*t*) = *h*([*x*,*y*]_0_, *t*) – *h*([*x*,*y*]_0_, *t*_0_). The accuracy of the determination of vertical position change equals the precision of ±0.5 nm. The precision was determined by the standard deviation of height changes measured at different areas inside the cavity. The growth rate is calculated from the slope of the vertical position change, d*z*/d*y* = dΔ*z*/d*t*.

### Thermodynamics and Kinetics of Calcite Growth

The saturation index Ω is related to the chemical potential μ_s_ of the solution
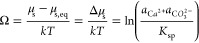
3where  and  are the ion activities, *kT* is the Boltzmann constant times temperature and μ_s,eq_ = μ_c_(*P* = 0) is the chemical potential of the solution when it is in equilibrium with it is unstressed crystal phase. Teng et al.^25^ have proposed that the solubility product *K*_sp_ = 10^–8.54^ corresponds to the experimental conditions when spirals on the 101̅4 surface stopped growing. We have used PHREEQC^26^ to calculate Ω.

A normal stress, or (load bearing contact) pressure, *P*_c_ on a solid surface contributes with *P*_c_*v* to the chemical potential of the crystal, Δμ_c_ = μ_c_(*P*_c_) – μ_c_(*P* = 0) = *P*_c_*v*, where *v* is the molecular volume of the solid.^27^ Thus, the chemical potential difference between the crystal in the load bearing contact and the solution
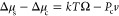
4drives either growth (when Ω*kT*/*v* > *P*_c_) or dissolution (when Ω*kT*/*v* < *P*_c_). The thermodynamic limit to the crystallization pressure is therefore *P*_c_ = Δμ_s_/*v* = Ω*kT*/*v*.

Since *kT*/*v* = 66 MPa one finds that a solution with saturation index of 0.44 is in equilibrium with a calcite surface subject to a pressure of *P*_c_ = 29 MPa. This is the thermodynamic limit of the crystallization pressure at saturation index 0.44. We may also calculate that the disjoining pressure of 0.5–5 MPa in the contacts amounts to reducing the driving force for growth, Ω – *P*_c_*v*/*kT* by 1.7–17% from 0.44 to 0.43 and 0.38, respectively. We have shown that in this range of saturation indices the purely kinetic growth rate constant (no diffusion limitation) is independent of saturation index^20^ and the kinetic contribution to the growth rate will therefore only be reduced by 1.7–17%.

## Results and Discussion

### Experimental Results

We have succeeded to nucleate and grow calcite crystals attached to the deformable membrane in several experiments. We have proceeded by increasing the control pressure to form a load bearing calcite–glass contact. All experiments have given qualitatively the same results but we focus here on the experiment where the contact stresses could be determined quantitatively and thereby be analyzed properly.

While bringing the crystal into contact with the glass, the flowing calcium carbonate concentration is kept at =0.05 mM and the saturation index Ω = 0. At time *t* = 0, the crystal is brought into contact with the glass surface with a fluid control pressure *P*_2,0_ = 20 kPa. The flowing fluid composition is also changed to = 0.8 mM, Ω = 0.44 at time *t* = 0. Figure 2 shows the evolution of the confined crystal surface after it has been brought in contact with the glass surface. The crystal grows outward, changing the area *A* of the crystal parallel to the glass. The crystal also grows downward in the load bearing contacts (perpendicular to the glass surface and image plane) pushing the crystal upward against gravity and the applied force of the membrane.

At time *t* = 0 the calcite surface was flat (*h* = 30 ± 10 nm, Figure 2A) and glass-calcite contact pressure *P*_c,0_ = 20 ± 10 Pa (see Figure S1 in Supporting Information). The calcite surface was confined and the Ca^2+^ and CO_3_^2–^ diffusion is limited in the confined solution film. The diffusion of ions into the film is only sufficient to support growth at the outer rim of the surface (greyscale in Figure 2A–L) and the inner region of the confined surface, [*x*,*y*]_0_ has no supersaturation, Ω = 0, no growth and is left as a cavity indicated by blue tint in Figure 2 A-L. We have previously explained this confined growth transition in detail.^19,22^ At the growth rim the growth continues, pushing the crystal upward.

**Figure 3:**
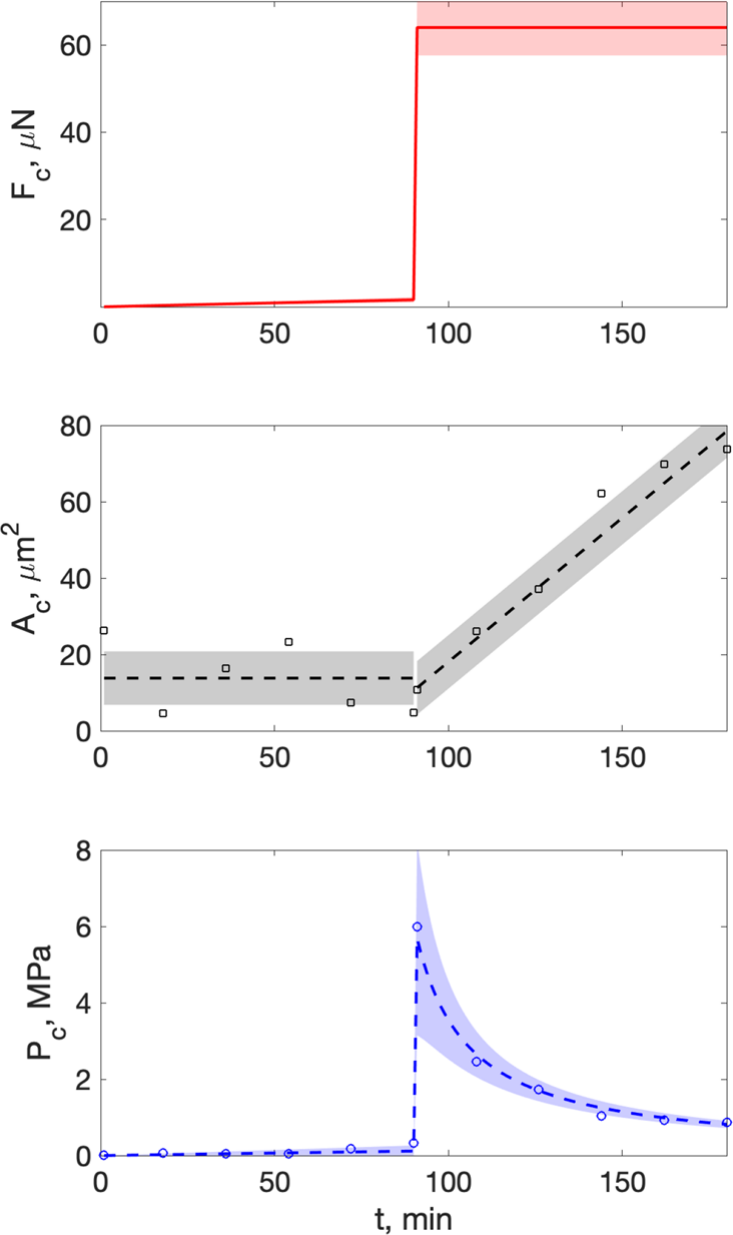
Membrane force, contact areas, and contact pressures. Top: Imposed force, *F*_c_, from membrane on crystal due to control fluid pressure as a function of time. Middle: Load bearing contact area *A*_c_ is the area of crystal-glass distance smaller than 20 ± 10 nm as measured in the interferometry images (see Figure 2) and smooth fit of *A*_c_ (dashed line). Bottom: Contact pressure, *P*_c_ = *F*_c_/*A*_c_ in load bearing contacts between calcite and glass support as a function of time. Shaded areas around lines represent standard errors propagated from the error sources discussed in the text. When no shading is visible the error is smaller than the thickness of the line.

We have used the RICM images in Figure 2 to measure the contact area, *A*_c_(*h* = 20 nm) between the calcite and the glass support (see Figure 3). The contact pressure is then calculated as *P*_c_ = *P*_c,0_ + (*A*_m_Δ*P*_2_ – *k*Δ*z*)/*A*_c_, where the differences Δ are with respect to time *t* = 0 (see Experimental Section and the first two sections of Supporting Information). In the first 90 min the contact area fluctuates as new contacts take over for old contacts and because the crystal pushes upward the mean contact pressure increased from 20 ± 10 Pa to 0.3 ± 0.1 MPa.

After 1.5 h growth with saturation index Ω = 0.44, the pressure in the upper channel is increased by Δ*P*_2_ = 10 to 30 kPa, which caused a change in the contact pressure from 0.3 ± 0.1 to 6 ± 0.6 MPa. The saturation index of the fluid is kept constant. The upward growth rate then comes to a halt and the contact area grows as the growth rim accommodates to the glass surface and the outer edge of the crystal continues to grow. The contact pressure reduced toward 0.8 ± 0.1 MPa as the contact area *A*_c_ grew.

**Figure 4:**
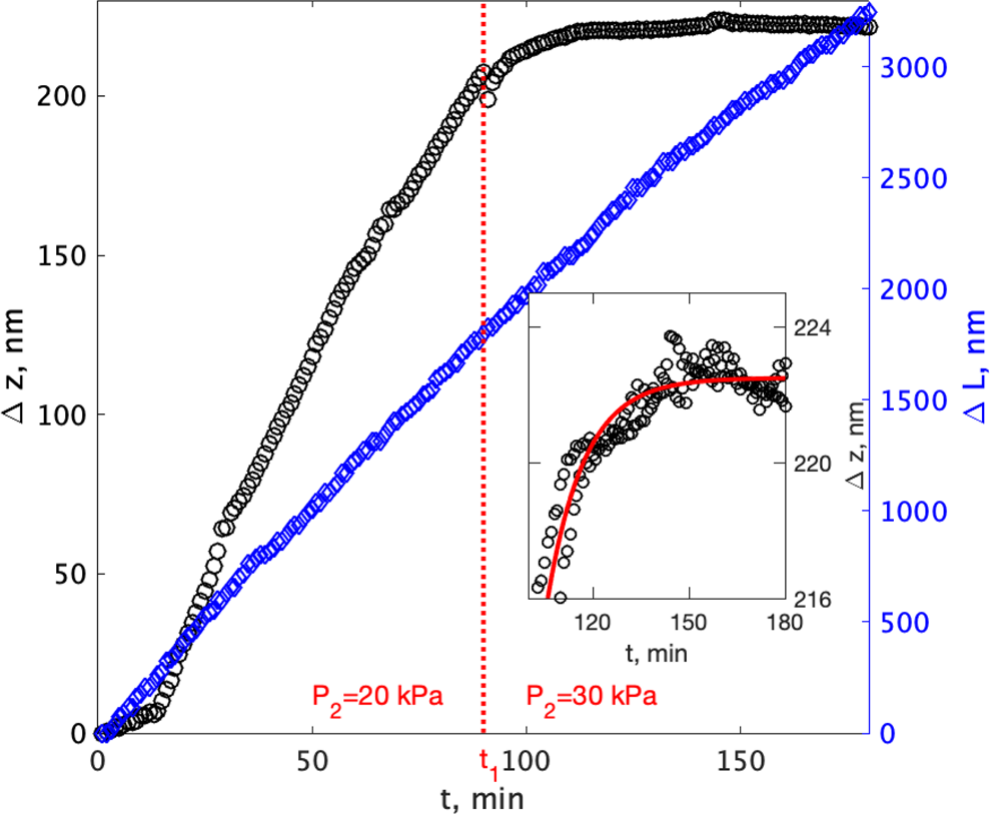
Calcite growth rate in all directions before and after fluid pressure, *P*_2_, increase at *t*_1_ = 90 min. Blue curves: lateral growth, no effect on rate of changing fluid pressure *P*_2_. Black data points: vertical growth comes to halt when pressure is increased. Inset: exponential decay of rate after pressure increase measured at two different areas in the middle of the crystal. The standard deviation of the height measurement during the last 40 min is 0.5 nm.

During the 3 h (180 min) with high saturation index, Ω = 0.44, the outer rims of the calcite crystal grew at a constant rate as can be seen in Figure 4. The upward growth shown in the same figure, however, goes through three distinct phases: The first 13 min the confined surface grows to accommodate the contact and tilting the crystal slightly, then during the period 13–90 min there is a constant upward growth rate of 2.6 nm/min. If the load were held constant the upward growth rate would have remained constant as seen in previous experiments (see Figure 4 in ref (22)). At *t*_1_ = 90 min the pressure increase pushes the crystal 8 nm downward and then the vertical growth rate instantly changes from constant to exponentially decaying and eventually comes to a complete halt. We continued to let the crystal grow under this load for 12 h more, but there was no further growth upward within the accuracy (1 standard deviation during the last 40 min, see inset in Figure 4) of ±0.5 nm. We can therefore conclude that the growth rate is smaller than 0.04 nm/h which equals 0.35 μm/yrs or 35 cm/Ma. Relative to the height of the crystal this growth rate corresponds to a strain rate of 10^–9^ s^–1^. Thus, even though we are below the detection limit of a very accurate technique the growth rate and strain rate could still be considerable on a geological time scale. This result requires us to pose the question: does the change in growth rate of more than a factor 4000 signify a dramatic slowing down of the crystal growth (working against a force) or does it signify a complete stop?

**Figure 5:**
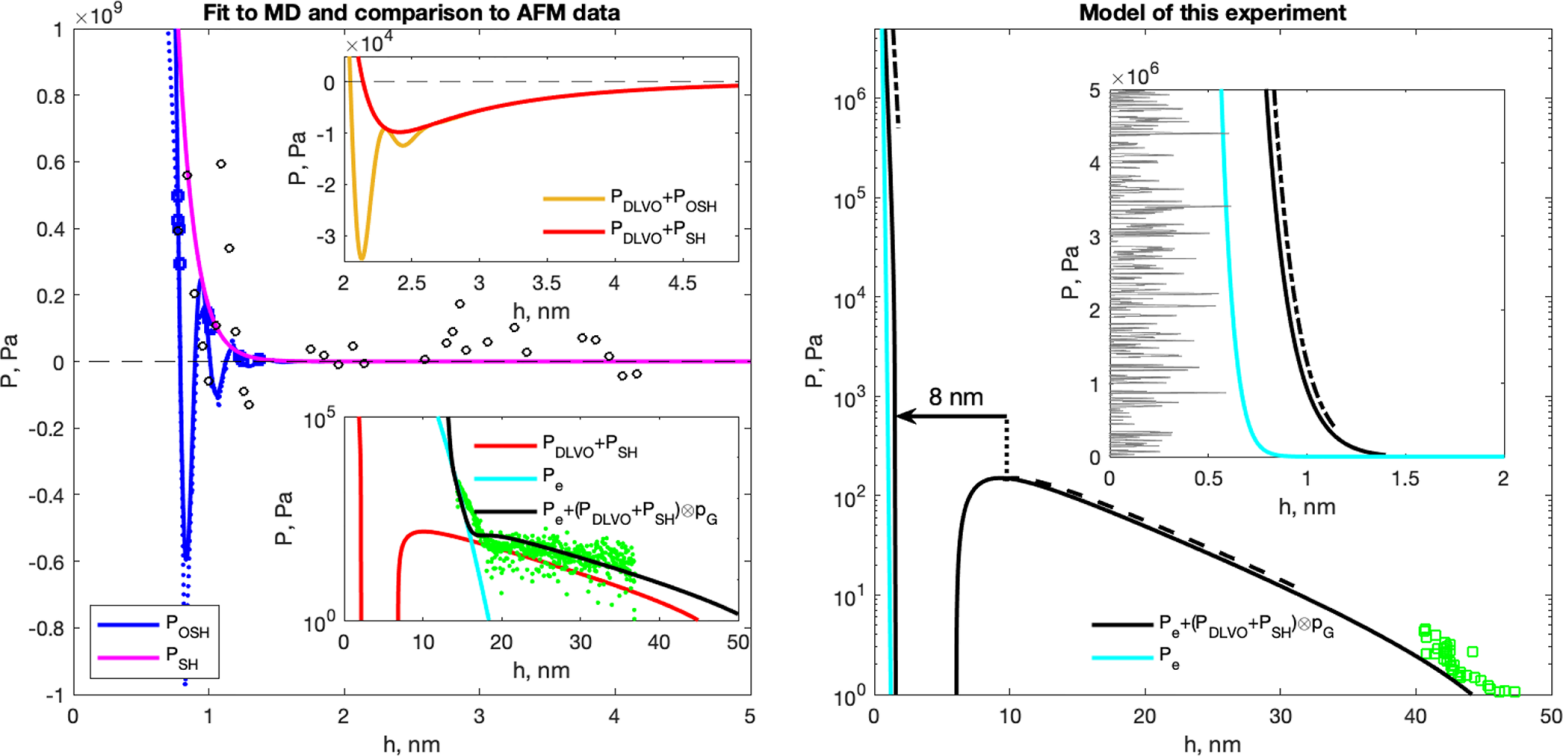
Disjoining pressure data and models for pure water in calcite and glass interfaces. Left: Simulation data (blue dots and squares,^28^ black circles^29^) for flat calcite surfaces and models fitted to the data (blue line: full fit, *P*_OSH_, magenta line: fit to repulsive part of MD data, *P*_SH_). Left, upper inset: Small amplitude, long-range attractive well from DLVO theory outside range of recent MD data.^28^ Left, lower inset: Green dots: experimental data for rough, glassy silica surfaces on atomically flat calcite surfaces.^24^ Black line: Parsons model with σ = 3 nm, cyan line: contact part of Parson model, red line: *P*_DLVO_ + *P*_SH_ as in upper inset. Right: Disjoining pressure model for load bearing contacts in this experiment: atomically flat calcite on rough glass with σ = 0.2 nm (black line). Green squares: experimental data from ref (22). Cyan line: contact part of Parson model. Gray: illustration of roughness with σ = 0.2 nm. Black dashed line: Pressure–distance range of this experiment before pressure increase at 90 min. Black dashed-dot lines: Pressure–distance range of this experiment after pressure increase at 90 min. As indicated by the arrow, increasing the pressure above 250 MPa causes an 8 nm jump in *h*.

Can the change in the gap, Δ*h* = 8 ± 2 nm, reduce the mass flow rate into the confined region enough to explain the data? If the rate of vertical growth is limited by diffusion
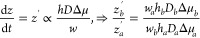
5where *D* is the diffusion coefficient, Δμ is the chemical potential difference between the solution and the crystal in the contacts, *w* is the width of the growth rim and indices *b* and *a* signify before and after. With a change from *h*_*b*_ = 10 nm to *h*_*a*_ = 2 nm, *h*_*b*_/*h*_*a*_ = 5 (see Figure 5) and the diffusion coefficient changes *D*_*b*_/*D*_*a*_ = 2.5 (see Figure S4 in Supporting Information). The width of the growth rim of *h* < 20 nm as in Figure 2 changes by a factor *w*_*a*_/*w*_*b*_ = 4. The average change in contact pressure is *P*_c*b*_ = 0.3 MPa to *P*_c*a*_ = 6 MPa which gives Δμ_*b*_/Δμ_*a*_ = 1.2 and the total change in vertical growth rate is only *z*_*b*_^′^/*z*_*a*_^′^=60. The observed growth rate is reduced by at least a factor 4000 but the reduction of the diffusion due to confinement can only account for only a factor 60. This means that some other mechanism is needed to explain a growth rate reduction of a factor 80 or more.

### Model Results

In this section we combine experimental data and simulation data, with a theoretical model that accounts for the forces between rough surfaces, to build a new model to describe the disjoining pressure between calcite and a rough glass surface. The disjoining pressure model is made up of the following main components: (1) van der Waals attractive interaction, (2) double layer repulsion (3) short distance steric repulsion (4) oscillatory forces due to water layering 5) accounting for surface roughness 6) elastic forces due to contacting asperities. The disjoining pressure is related to the interfacial energy per unit area, *E* as *P*(*z*) = −d*E*(*z*)/d*z*.

Diao and Espinosa-Marzal^24^ have performed high precision AFM measurements of forces between rough silica (SiO_2_) beads and cleaved (101̅4) calcite surfaces. This is very similar to our rough glass (SiO_2_) and (101̅4) calcite growth surfaces. We therefore use the Hamaker constant of the van der Waals interaction and the double layer repulsion parameters they derived from their data. The combination of the two interactions, the so-called DLVO model that we here call *P*_DLVO_ can be found in the Supporting Information of Diao and Espinosa-Marzal.^24^

At surface separations less than 3 nm, steric repulsion can be modeled as an exponentially decaying function and the oscillatory forces due to water layering is a multiplicative factor.^30^ The oscillatory force is due to the tendency of water to form layers at most solid interfaces. The short wavelength of the oscillations is due to the lateral order induced by the calcite surface. There is no data available for calcite–glass surfaces that are flat enough to reveal these interactions. We will therefore use calcite–calcite surfaces as the best starting point to model interactions between atomically flat calcite–glass surfaces. We have used the MD data of Brekke-Svaland and Bresme^28^ that shows oscillatory steric/hydration forces (OSH) between two flat calcite surfaces in water. We have fit their pressure to *P*_DLVO_ + *P*_OSH_ where *P*_OSH_ is a periodic, exponentially decaying function similar to the suggestion by Israelachvili^30^
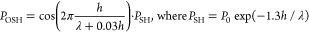
6λ = 0.162 nm is the period of the oscillations as determined by the fit and *P*_0_ = 5 × 10^11^ Pa. The fit and the data from refs (28) and (29) (shown in blue^28^ and black^29^) is shown in the left plot in Figure 5.

The top inset on the left-hand side compares the depth and width of the attractive regions due to DLVO and water layering. One observes that the contribution of *P*_OSH_ is only important at *h* < 2.5 nm and especially at around 1 nm.

The next step is to take into account the silica surface roughness. We use the rough surface model of Parsons et al.^31^ with an RMS roughness σ. The resulting disjoining pressure model can be expressed as
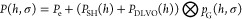
7where ⊗ represents the convolution with the Gaussian height distribution *p*_G_(*h*, σ) with σ the RMS roughness and *P*_e_ is a contribution from elastic contacts between asperities of the surfaces. The distance between the surfaces *h* is now an average distance and the total pressure *P*(*h*, σ) averages out the local attractive and repulsive interactions. We compare the resulting model *P*(*h*, σ) to the AFM data for rough silica spheres on calcite,^24^ featuring a steep repulsive part *P* > 100 Pa and a long-range tail of *P* = 10–100 Pa (see bottom inset on the left side of Figure 5). The only adjustable parameter required to obtain the black curve that passes through the AFM data, was the RMS roughness, σ, of the silica sphere. We used here σ = 3 nm, close to the value reported by the authors, σ = 2 nm.^24^ One observes that even though there are local attractive interactions between the surfaces, the net interaction is always repulsive. We have displayed a model rough surface with σ = 3 nm to illustrate the surface that is integrated over in the final model.

The same model is then used to predict the disjoining pressure curve for our experiments with glass roughness σ = 0.2 nm (see right-hand side of Figure 5). The model fits very well with our previously published data (green squares). The model predicts the range of average distances between the two surfaces in the first 90 min (10–250 Pa ⇒ 10–30 nm) and in the last 90 min (5 × 10^5^–5 × 10^6^ Pa ⇒ 2.4–2.6 nm). This corresponds well with the observed jump of 8 ± 2 nm when the pressure was increased. In Figure 5, right-hand we have in gray displayed a model rough surface with σ = 0.2 nm. One observes that there are no solid–solid contacts between the two surfaces, but there are several points where the local distance is around 0.8 nm. For comparison, an atomic layer of calcite is 0.32 nm.

## Discussion

We have shown that when the pressure at the confined interface is increased enough to establish close proximity (*h* ≈ 2.5 nm), the confined vertical motion of the crystal is reduced by at least a factor 4000 and apparently stops completely. We have observed the same arrest of vertical motion in many other experiments.^19,22^ The reduced diffusion transport of ions to the confined surface and the change in thermodynamic driving force or growth rate kinetics may all together account for a reduction by a factor of 60 only. If there are only repulsive forces between the surfaces, whatever *A*_*c*_, one expects the vertical growth rate to be reduced immediately upon pressure increase and then the growth rate should increase again as *A*_*c*_ grows, which is the opposite of the exponential halt of vertical growth rate that we observe.

**Figure 6:**
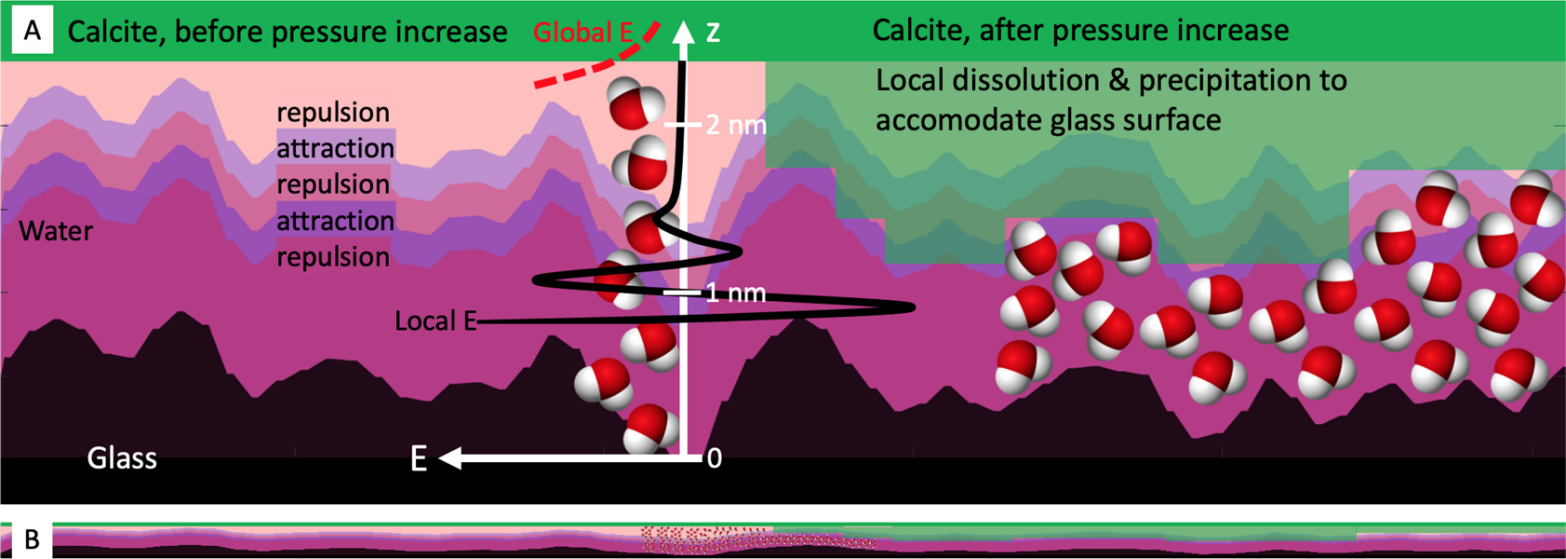
Adhesion arrests repulsive crystal growth. (A) Twenty times vertically exaggerated. (B) Equal vertical and horizontal scale. Calcite (green) is pressed against a rough glass (black) surface with water (purple hues) in between. More reddish purples signify local repulsive energy and more blueish purples signify local attractive energy between calcite and glass due to the local water ordering. The black whole drawn curve is the local energy *E* per unit area calculated for flat surfaces (like the pressure *P* = −d*E*/d*z* displayed on left side of Figure 5). The red dashed curve is the global energy taking roughness into account (like the pressure displayed in the inset of the right-hand side of Figure 5). On the left-hand side of the figure we display the situation in the first part of the experiment when the calcite crystal grows and is pushed upward. The entire calcite surface is subject to a repulsive force slowly varying tangentially to the surface. On the right-hand side of the figure we display the calcite surface after pressure increase and arrest of crystallization. The local calcite surface height is mostly adjusted through dissolution and precipitation to achieve a local low energy state with 3–4 well ordered layers of water. The energy barrier to further crystallization is therefore very high. Consequently, maximum crystallization pressure is not dictated by supersaturation as predicted by thermodynamics but by surface interaction parameters like the interaction energy and roughness.

To explain the arrest of the vertical motion we propose a new mechanism. Figure 6 sketches a molecular interpretation of the processes at play between the rough inert glass surface and the reactive calcite surface that we have modeled in Figure 5. Figure 6A shows the vertical scale at least 20X the horizontal scale, whereas Figure 6B is just included to show that in reality the length scale of height variations of the glass surface is long compared to the thickness of the water layer. When the surfaces are pressed together by more than 1 MPa the mean distance between a rough (σ = 0.2 nm) and a flat surface is 1–3 nm depending on the nature of the fluid and charges on the surfaces. However, local regions of the calcite surface are closer or more distant to the glass surface. Due to the ordering of the confined water, different regions of the surfaces will experience either local adhesion or repulsion. In addition to the chemical potential Δμ of supersaturation, there is a local free energy difference driving crystal growth in the regions where the growth results in increased adhesive energy. Local pressures exceeding *P*_c_ = Δμ/*v*, will drive local dissolution. Consequently, once the surfaces are brought into sufficiently close contact, local dissolution and growth will reshape the calcite surface to fit the glass surface, leading to a maximization of the local regions experiencing an attractive interaction, at typical separations of 3–4 water layers. (The exact number of water layers does not matter to the argument.) Growth on this surface is inhibited by the local energy cost that far exceeds the Gibbs free energy of supersaturation. Consequently, crystal growth ceases and the surfaces adhere instead of being pushed apart. If the surfaces do not have appreciable adhesive regions, the calcite surface may still locally deform by dissolution/precipitation to approach the other surface. This will significantly slow down diffusion and crystal growth without the surfaces adhering. The proposed mechanism will depend on the surface roughness, the hydrophilic/phobic nature of the surfaces and the fluid composition. These are interactions that can be modeled^30^ to predict the maximum crystallization pressure.

It should be noted that the proposed mechanism is valid for surfaces that are globally repulsive in the sense that the solid–solid interfacial energy is larger than the sum of the solid–liquid interfacial energies. If the solid–solid interfacial energy is lower than the sum of the solid–liquid interfacial energies the solids can form solid contacts and the crystallization will be immediately arrested by the solid–solid adhesion.^32^ This mechanism can not explain the exponential slowing down of the vertical motion unless one invokes some process of continuously forming and rupturing of solid contacts.

It has been demonstrated that liquid ordering is important during crystal growth^33^ and that crystallization can take place in local regions. The crystallization is correlated with the observation of negative and positive disjoining pressures, which may change on very short length scales (nano and subnanometer distances).^34^ A recent study of contact formation using Kinetic Monte Carlo (KMC)^35^ shows that growth of local contacts is enhanced by an attractive interaction energy of the same order as that created by the ordering of 3–4 confined water layers (see KMC section in Supporting Information).

SFA and AFM experiments demonstrate that roughness is important for short time adhesiveness and dissolution– precipitation processes in the confined region.^24,36−39^ Systematic variation of contact time should allow a better understanding of the adhesion forming mechanism that we propose here. Indeed, we evidenced an analogous calcite growth mechanism in the SFA experiments with reactive calcite surfaces growing against a mica substrate (see Figure S5). These SFA results indicate that the growing calcite asperities become locally smoother, leading to the stronger adhesion between calcite and mica with time.

The proposed mechanism is closely related to adhesion between reactive solids and resembles the molecular scale processes proposed for crystal agglomeration.^40,41^ Experiments on the interactions between reactive surfaces in the surface forces apparatus (SFA),^37,39^ with AFM^24,36,38^ and slide-hold-slide friction^42^ all show that the adhesion between two surfaces depends on the fluid present, the force applied and time spent holding the surfaces together before pulling them apart or sliding.

The proposed mechanism is also consistent with recent experimental observations that showed that the limit to crystallization pressure is related to the disjoining pressure and not to the thermodynamic limit pressure.^9,11^ The proposed mechanism can be thought of as “microfracture healing” without forming covalent bonds, only weak water-film-mediated “bonds”. This mechanism can explain several experimental observations of reactive interfaces developing strength with time: fracture healing,^43^ cement setting^44^ and fault gouge strengthening.^42,45^

Recently, it has been demonstrated that the crystallization pressure of NaCl on glass is reduced exponentially with supersaturation even though the thermodynamic limit *increases* with supersaturation.^9^ The authors argued that the crystallization process was arrested once the fluid film reached a thickness of about 1.5 nm. Our experimental and modeling study on the nanoscale explains the mechanism how crystallization pressure is arrested at fluid film thicknesses of 3–4 water layers. We also demonstrate that modeling of the surface forces including roughness may predict the limit of crystallization pressure.

A systematic evaluation of the proposed mechanism can be performed both by Kinetic Monte Carlo (KMC)^35,46,47^ and experimentally combining optical imaging of the contacts with AFM experiments.^24,36^ The existence and effectiveness of the proposed mechanism depends crucially on the roughness and surface forces. These are parameters that can easily be varied experimentally and in KMC. Molecular simulations of hydrated crystals like mirabilite and alum may reveal whether their large crystallization pressures and damaging properties^1,2,15^ are due to qualitative differences in water structure, adhesion and diffusion as compared to nonhydrated crystals like CaCO_3_, NaCl^9,16,18^ NaClO_3_.^19^

## Conclusions

A new experimental technique to control and image crystal growth in nanoconfinement has been developed and applied to calcite and shown that displacement by crystallization pressure is arrested at pressures well below those corresponding to the thermodynamic limit. Existing simulation and AFM experimental data have allowed us to build a robust model to rationalize the disjoining pressure in our system and thereby calculating the absolute distance between the surfaces. Our findings are consistent with recent experimental observations that suggested that the limit to crystallization pressure is related to the disjoining pressure and not to the thermodynamic limit pressure.^9,11^ Our detailed experiments and modeling indicate that the mechanism responsible for the arrest of crystal growth is connected to contact healing processes, which create strong but noncovalent adhesion between surfaces confining nanoscale films containing 3–4 layers of water molecules. The new mechanism is strongly dependent on the nature of the surfaces, the roughness and the fluid composition. Understanding this mechanism will allow prediction of the limit between damage and adhesion by crystallization in many systems in Earth and materials sciences.

## Supporting Information Available

Additional figures, text, and equations (PDF)
Timelapse movie of nanoconfined calcite surface evolution associated with Figure 2 (AVI)

## Supplementary Material

la2c01325_si_001.pdf

la2c01325_si_002.avi
