# Supplementary material for: Limits to Crystallization Pressure
Source: Langmuir. 2022 Sep 9;38(37):11265–73. doi: 10.1021/acs.langmuir.2c01325 (PMC9494941; doi:10.1021/acs.langmuir.2c01325)
Supplement: Supplementary file 1 — la2c01325_si_001.pdf [file la2c01325_si_001.pdf]

Limits to crystallization pressure
Supporting Information

Lei Li,[†,‖] Felix Kohler,[†,⊥] Joanna Dziadkowiec,[†] Anja Røyne,[†] Rosa M. Espinosa Marzal,[‡] Fernando Bresme,[¶] Espen Jettestuen,[§] and Dag Kristian Dysthe[*,†]

†*Physics of Geological Processes (PGP), The NJORD Centre, Department of Physics, University of Oslo, PObox 1048 Blindern, 0316 Oslo, Norway*

‡*Environmental Engineering and Science, Department of Civil and Environmental Engineering, University of Illinois at Urbana–Champaign, Urbana, IL 61801, USA*

¶*Department of Chemistry, Molecular Sciences Research Hub, Imperial College, W12 0BZ, London, United Kingdom*

§*Norce Research, Essendropsgate 3, 0368 Oslo, Norway*

‖*Present address: College of Physics and Optoelectronic Engineering, Shenzhen University, Shenzhen 518060, China*

⊥*Present address: Expert Analytics, Møllergata 8, 0179 Oslo, Norway*

E-mail: d.k.dysthe@fys.uio.no

Force applied to crystal

When the calcite is located and growing on the PDMS membrane in the lower channel, we start to increase the control pressure P_2 slowly until a corner of the calcite reaches the cover glass at $P_2 = 19$ kPa as shown in Figure S1. The calcite is at this pressure tilted along the white line with a maximum distance of 560nm. In order to bring the calcite surface parallel to the cover glass surface, P_2 is increased to 20 kPa as shown in Figure S1. The average

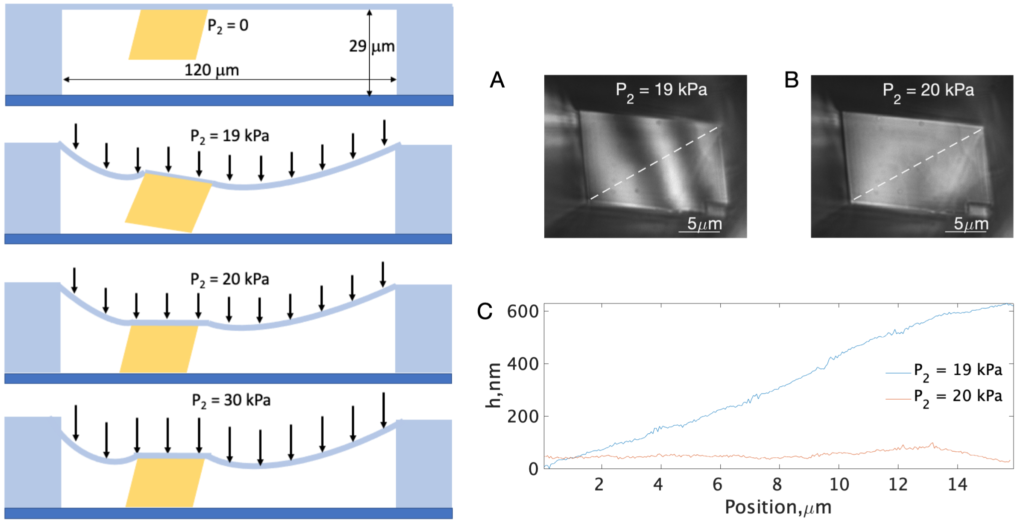

Figure S1: Deformation of membrane and force application on crystal. Top: Initially the crystal is suspended underneath the PDMS membrane. The pressure is increased to 19 kPa where one corner of the crystal touches the glass. Further pressure increase to 20 kPa brings the whole crystal surface in contact with the glass. Final pressure increase to 30 kPa does not move crystal, only increases the force between crystal and glass. Bottom part: A and B shows RICM images of crystal brought into contact with glass. C: Distance profile between glass and crystal along whate dashed lines in A and B.

distance along the white line is then 57 nm and the average distance h of the whole surface is 30 nm. As documented previously[1] the disjoining pressure P_c between calcite and a glass surface at distance $h = 30$ nm is $P_c = 20 \pm 10$ Pa. Due to force balance the force transmitted from the membrane to the crystal is thus the contact pressure P_c times the area A_c of the crystal: $F_{c,0} = P_c A_c \approx 20$ Pa$\times 370$ μm$^2 \approx 7$ nN.

We do not know the thickness of the crystal, but it is in the range of 10-20 μm, thus the initial vertical displacement of the crystal on the membrane is between 10 and 20 μm and we will use $z_0 = 15 \pm 5$ μm. We can assume that the membrane behaves elastically, this means that while the crystal does not touch the glass $A_m P_2 = kz$, where k is a constant of elasticity and A_m is a representative membrane area. The representative membrane area A_m is the area of the membrane closer to the crystal than the edges of the channel. This has been found to be $A_m = 6400 \pm 640$ μm^2 by performing a Euclidean distance transform followed by a watershed transform on an image of the crystal in the channel. The membrane

stiffness is then estimated to be

$$k = \frac{A_m P_2}{z} = \frac{6.4 \cdot 10^{-9} 1.9 \cdot 10^4}{1.5 \cdot 10^{-5}} = 8 \pm 3\text{N/m}. \tag{S1}$$

Once the crystal touches the glass the force on the crystal-glass interface is thus

$$F_c = A_m P_2 - kz. \tag{S2}$$

Taking the crystal initially flat on the glass as our reference state, $z_0 = 15 \pm 5$ μm, $P_{2,0} =$ 20 kPa, $F_{c,0} = 7 \cdot 10^{-9}$ N, we can calculate the force from the crystal on the glass surface as

$$F_c = F_{c,0} + \Delta F_c = F_{c,0} + A_m \Delta P_2 - k\Delta z, \tag{S3}$$

where $\Delta P_2 = P_2 - P_{2,0}$ and $\Delta z = z - z_0$. When the control fluid pressure is increased to 30 kPa ($\Delta P_2 = 10$ kPa) the crystal has grown vertically $\Delta z = -0.2$ μm and the crystal-glass force is $F_c = F_{c,0} + A_m \Delta P_2 - k\Delta z =$7 nN + 1.6±0.6 μN + 64±6.4 μN ≈ 66±7 μN.

Load bearing contact area

The load bearing area of contact A_c as function of time and threshold fluid film thickness h is displayed in Figure S2. The forces on areas with $h < 50$ nm and $h < 10$ nm are $F > 1$ Pa and $F > 100$ Pa respectively. The accuracy of the determination of h from intensity is ±10 nm due to small variations in illumination. One observes that whatever the threshold there are certain features that stay the same:

- At low force the contact area varies with time as new contacts form and others disappear
- Between $t = 90$ and 91 min all areas of contact increase when the force is increased and the crystal is pushed 8 nm closer to the glass.

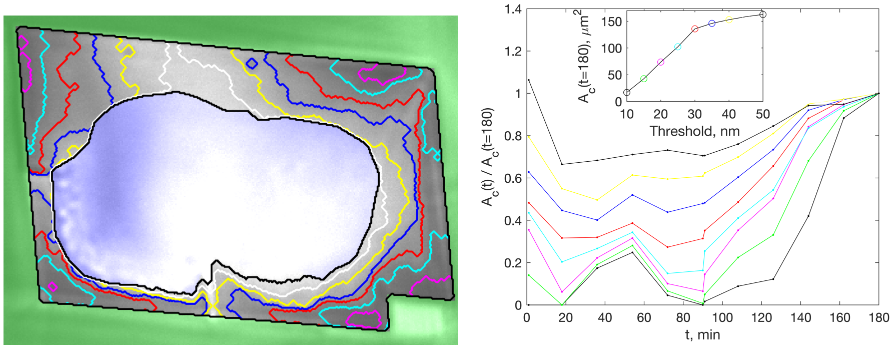

Figure S2: **Load bearing contact area A_c as function of fluid film widths h threshold.** Bottom: RICM image at $t = 91$ min thresholded at intensities corresponding to different fluid film widths, h and the contours are drawn with the same color code as in inset of top figure. Top: The area A_c of the crystal closer to the glass (lower intensities) than the threshold h normalized by final contact area is calculated in each of the 12 images (at times 1-180 minutes). The maximum area max(A_c) for each threshold is plotted in the inset and the colors of the symbols correspond to the colors of the curves for each threshold.

- After the force is increased the contact area grows quickly at first and the growth slows down after 144 min.
- The contact area increases monotonically with the threshold.

We have also included the histograms of fluid film thicknesses at the growth rim, calculated from the RICM images in Figure 2.

Calcite - silica interactions

We combine recent experimental and molecular dynamics data for pressures and diffusion in confined calcite-calcite and calcite-silicate interfaces. We demonstrate how to combine data from experiment and simulation to calculate effective disjoining pressure and diffusion and thereby obtain predictions for deformation rate versus confining pressure.

Recently we have succeeded in measuring surface forces between calcite surfaces and between silica surfaces and calcite surfaces. The last 20 years has also allowed molecular simulation of calcite and silica surfaces with increased refinement and detail. It is not straight forward, however to compare molecular dynamics (MD) simulations, atomic force microscopy

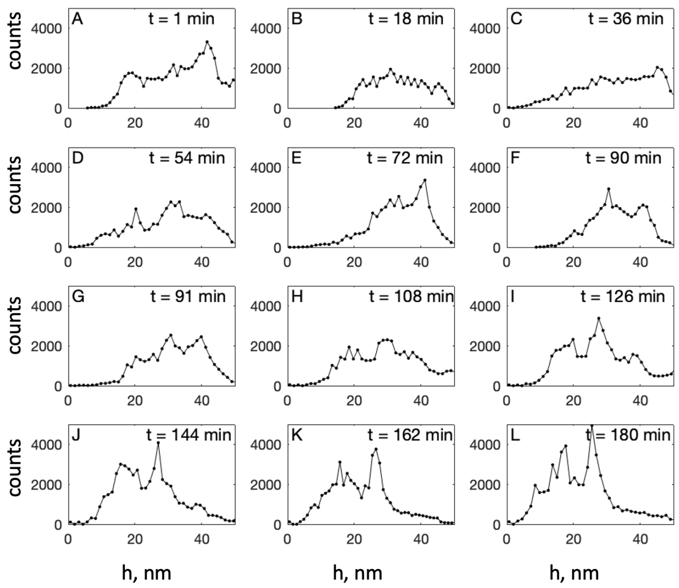

Figure S3: Histogram of fluid film thickness along the growth as function of time.

(AFM) and surface forces apparatus (SFA) measurements. The individual experiments and simulations are also not easily applied to real problems like colloidal aggregation, material strength, reactivity in confinement, etc.

The aim of this section is to combine experimental data, molecular simulation and theory and use interaction data from MD and AFM to construct calcite-calcite and calcite-silicate interaction potentials, forces or pressures for flat rough surfaces. In addition we estimate the effect of confinement on diffusion as function of stress and surface roughness.

Interaction energies, forces, pressure, roughness and geometry

The oscillatory and hydration-steric forces of atomically flat calcite and silica surfaces are as of yet only available from molecular simulation. We can use insights from and models based on measurement of mica-mica forces to extrapolate and add to the DLVO forces. In order to compare this to force measurements on calcite and silica surfaces we need to take the roughness of the surfaces into account.

Disjoining pressure from DLVO theory

The disjoining pressure P from the DLVO theory is calculated according the supplementary information of Diao and Espinosa-Marzal:[5]

$$P = -\frac{du(h)}{dh}, \tag{S4}$$

where the energy of interaction between two planar surfaces is composed of the van der Waals energy $u_{vdW}(h)$ and electric double layer energy $u_{EDL}(h)$:

$$u(h) = u_{vdW}(h) + u_{EDL}(h). \tag{S5}$$

The van der Waals energy is

$$u_{vdW}(h) = \frac{A}{12\pi h^2}, \tag{S6}$$

where $A = 6.95 \cdot 10^{-21}$ J is the Hamacker constant for the calcite-glass interface. The electric double layer energy is

$$u_{EDL} = 2\pi\epsilon_0\epsilon_\kappa \frac{2\psi_c\psi_s e^{-\kappa h} + e^{-2\kappa h}\left((2p_c - 1)\psi_c^2(2p_s - 1)\psi_s^2\right)}{1 - (2p_c - 1)(2p_s - 1)e^{-2\kappa h}}, \tag{S7}$$

where $\epsilon_0 = 8.85{\cdot}10^{-12}$ F/m is the permittivity of vacuum, $\epsilon = 80$ is the relative permittivity of water, the inverse Debye length is $\kappa = 1/\lambda_D$, the surface potentials of calcite $\psi_c = -12.8$ mV and glass $\psi_s = -6.3$ mV and the regulation parameters are $p_s = 0.88$ and $p_c = 0.62$.

Roughness

The free energy of interaction, $u(h)$, between two planar surfaces separated by an electrolyte of thickness h have many contributions. From a theoretical viewpoint one normally assumes that the interaction energy contributions from different parts of the solid surfaces and from different effects in the fluid are all additive. Then one can integrate over the shapes of the

surfaces to obtain the force $F(h) = dU(h)/dh$ between the two solids, where $U(h)$ is the free energy integrated over the surfaces. The force between two spheres of radius r_1 and r_2 is thus calculated to be[2]

$$F(h) = 2\pi \left(\frac{r_1 r_2}{r_1 + r_2} \right) u(h). \tag{S8}$$

This is called the Derjaguin approximation and is practical to compute forces between two spheres (for example colloidal particles), a sphere and a flat (atomic force microscope (AFM) with a spherical tip on a flat surface) or two cylinders (as in the surface forces apparatus (SFA)). The same assumption of additivity may be used for any surface shape as long as the radii of curvature of the surfaces are much larger than the range of $u(h)$.

Treating rough surfaces statistically Parsons et al[3] recently proposed performing the corresponding integral over interaction energies using the height probability distributions $p_i(z_i)$ of the two surfaces:

$$U(h) = \int_{-\infty}^{\infty} \int_{-\infty}^{\infty} dz_1 dz_2 p_1(z_1) p_2(z_2) u(z_1 - z_2 + h). \tag{S9}$$

This integration is sufficient when the surface roughness, $\sigma_i = (\int_{-\infty}^{\infty} dz_i z_i^2 p_i(z_i))^{1/2}$, is much smaller than the range of $u(h)$. When roughness is larger some points of the surfaces will contact and deform elastically or plastically. Parsons et al[3] proposed to treat the contacting asperities as Hertzian contacts with an effective asperity radius r_a. Assuming $p_i(z_i)$ to be Gaussian they can be combined into a single distribution $p(z)$ with variance $\sigma^2 = \sqrt{\sigma_1^2 + \sigma_2^2}$ and the Hertzian contact contribution, U_C, to the interaction between the surfaces is:[3]

$$U_C(h) = \frac{2E\sigma}{15\pi^{3/2}} \sqrt{\frac{\sigma}{r_a}}\, e^{-\frac{h^2}{2\sigma^2}} f(\frac{h}{\sigma}), \tag{S10}$$

where E is an effective Youngs modulus and f is a geometrical function.[3] Both the height probability distributions $p_i(z_i)$ and the typical asperity radius r_a may be estimated from AFM imaging of the surfaces. The total interaction energy, $U_t = U + U_H$, is the sum of the

contact and non-contact contributions.

The thermodynamically most useful measure of the surface interactions is the pressure $P(h) = dU(h)/dh/A$, where A is the macroscopic contact area of the surfaces.

Disjoining pressure model for rough silica on flat calcite

In order to combine molecular dynamics, theory and experiment we have combined the model fit in the previous section with experimental parameters and data from Diao and Espinoza-Marzal.[5] They reported that the roughness of the silica spheres was approximately σ =2 nm. To model this roughness we used a truncated normal distribution

$$n(z) = \begin{cases} \frac{1}{N} e^{-\frac{z^2}{2\sigma^2}}, & |z| < 2\sigma \\ = 0, & |z| > 2\sigma \end{cases}, \tag{S11}$$

where the $N = \int_{-2}^{2} dz e^{-\frac{z^2}{2\sigma^2}}$ normalizes n as a probability distribution. The pressures $P_{R,i}$ for the rough system are then

$$P_{R,i}(h) = \int_{-2\sigma}^{2\sigma} dz P_i(h - z) n(z). \tag{S12}$$

The elastic contact contribution to the pressure is found by differentiating equation (S10):

$$P_C(h) = \frac{2E\sigma}{15\pi^{3/2}} \sqrt{\frac{\sigma}{r_a}}\, e^{-\frac{h^2}{2\sigma^2}} \left(\frac{h}{\sigma} f(\frac{h}{\sigma}) - f'(\frac{h}{\sigma}) \right). \tag{S13}$$

The experimental data of Diao and Espinosa-Marzal[5] for 0.51 mM $CaCO_3$ solution that has been translated along the x-axis to coincide with the pressure model. The shift is justified because the AFM data has no intrinsic reference point of $x = 0$. In order to obtain a region of the model with a moderate slope corresponding to the data, the roughness and asperity radius were adjusted to $\sigma = 3$ nm and $r_a = 10$ nm.

We have also included data from Li et al[1] of a crystal lying on a glass surface with

roughness of about 0.2 nm. This data agrees perfectly with the roughness corrected pressure model presented here.

Diffusion

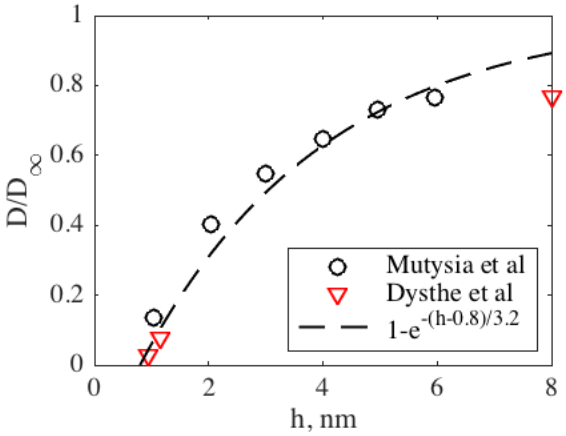

Figure S4: Self diffusion of water in calcite slit pores as function of the water film thickness, h.

Self diffusion of water in a calcite slit pore has been calculated by Dysthe et al[6] and Mutysia et al.[7] The interaction potential model used by Mutysia et al[7] is more evolved and adapted to a range of properties of calcite-water interfaces. A rough empirical fit to their data for water, D_w, yields $D_w/D_{w,\infty} = 1 - \exp(-(h - h_0)/\lambda)$ with h_0=0.8 nm and λ=3.2 nm and $D_{w,\infty}$ is the bulk diffusion coefficient. Figure S4 shows that the diffusion reduced coefficients $D_w/D_{w,\infty}$ calculated by Dysthe et al[6] with the simplified interaction potential at strongly varying temperatures agree well with the data of Mutysia et al.[7]

We are interested in the reactivity of a nanoconfined calcite interface and how it is limited by mass transport. The quantities of interest is then the interdiffusion coefficient of $CaCO_3$ that depends on the self diffusion coefficients of the Ca^{2+} and CO_3^{2-} ions. If we assume that the Stokes-Einstein relation $D = kT/6\pi\eta r$, where η is the viscosity and r is the particle radius, is valid for both water and ions. That is, the water self diffusion measurements reflect the change in viscosity of the fluid layer and that the mobility of all ions are inversely

proportional to this viscosity. Then the calcium carbonate diffusion coefficient, D_c should also be $D_c/D_{c,\infty} = 1 - \exp(-(h - 0.8)/3.2)$.

Diffusion in silica slit pores has been studied by Collin et al[8] and they found that similarly to calcite water diffusion is reduced by an order of magnitude for slit pores of 1 nm corresponding to 3 water layers.

One important aspect of a rough surface meeting a flat surface is that it leaves room for diffusion. At a high pressure like 1 GPa diffusion is very slow between two smooth, flat surfaces. For the rough silica - flat calcite contact at similar pressures only a small part of the surface has a distance $h < 0.8$ nm where diffusion is zero. Unless the reactive surface changes shape to conform with the rough surface, diffusion will continue and so will crystal growth.

Local contact formation modelled by Kinetic Monte Carlo

A recent study of contact formation using Kinetic Monte Carlo (KMC)[9] gives some insight into how local dissolution and growth form adhesive contacts between a reactive and a non-reactive surface. The model uses an exponential surface-surface repulsive energy $G_r = E_b \sigma_0 e^{-(h-h_0)/\lambda_D}$, where E_b is the depth of the energy minimum binding the two solids, σ_0 is the strength of the repulsive interaction, λ_D is the Debye length and h_0 is the position of minimum of the attractive potential $G_a = -E_b(3h + 64/h^6 - 7)/60, h \leq h_0$. The KMC simulation of an atomically flat non-reactive confining surface and a reactive surface with a loading pressure F_0/A can be compared to the experimental system in the following way:

There is a global loading force and a global disjoining force that balance as shown in the main text and in Figure S5**A**. In the experiments there are local asperities of the glass surface where fluctuations in the local height of the calcite may bring the two as close as 2 or 3 layers of water where there is attractive energy between the two surfaces (see Figure S5**C**). The depth of this energy minimum is mainly controlled by the ordering of the water

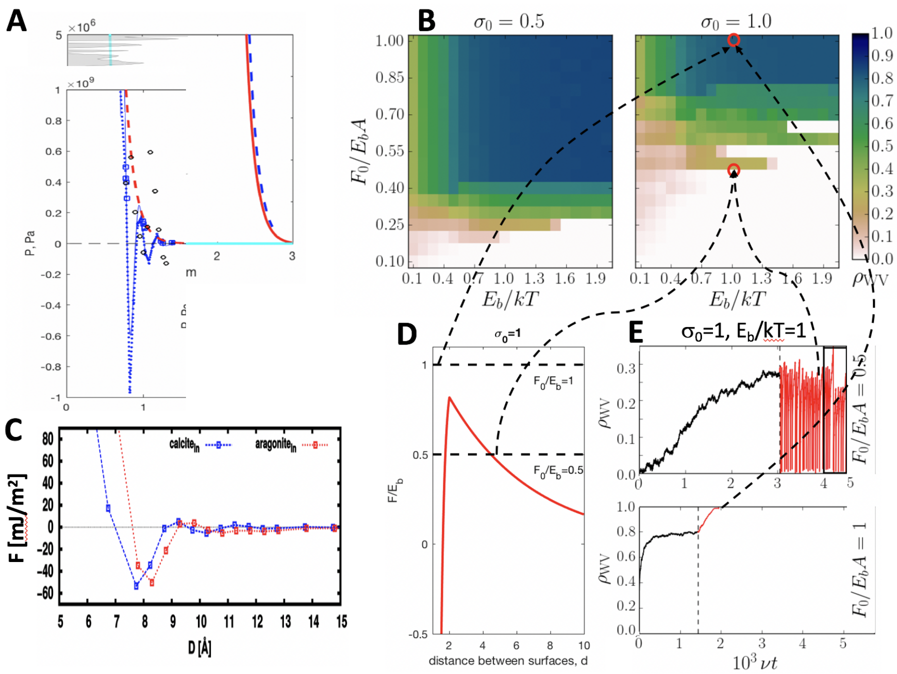

Figure S5: Growing contacts under global pressure and local attraction. **A:** Global repulsion between the calcite and the rough glass surface and the local oscillatory pressures (note different vertical scales). Image from this study. **D:** Repulsive and attractive pressure used in KMC model. **B and E:** Results of KMC simulations showing complete contact developing ($\rho_{WV} = 1$) when the load is larger than disjoining pressure peak and partial coverage and oscillatory behaviour when the load is smaller than the disjoining pressure and the equilibrium intersurface distance is 3 crystal layers. Images from.[9] **C:** Free energy of interaction between two flat calcite surfaces. The depth of the minimum due to ordering of 2 water layers is $F/kT = 2$. Figure reproduced from.[4]

layers between the surfaces. This is sensitive to the molecular structure of the surfaces and the relative positioning of the crystal lattices and for calcite-calcite F=-20 to -60kJ/m^2=-3.2 to -8.1·10^{-21}J per lattice site, thus $-F/kT = 0.8 - 2$. If the crystals are ordered such that the water layer ordering is completely broken the main minimum will be direct solid-solid contact (no water left in the contact).

This global repulsion, and local attraction resembles the KMC model as shown in Figure S5**D**. At lower loads the system can end up in an oscillating regime where contacts are formed and broken like we observe in the initial low load part of the experiments. As the load is increased there is a transition to full contact developing between the two surfaces. In our system this contact state may correspond to a local surface-surface distance of either

2-3 molecular water layers or to zero distance, that is solid-solid contact.

SFA experiments

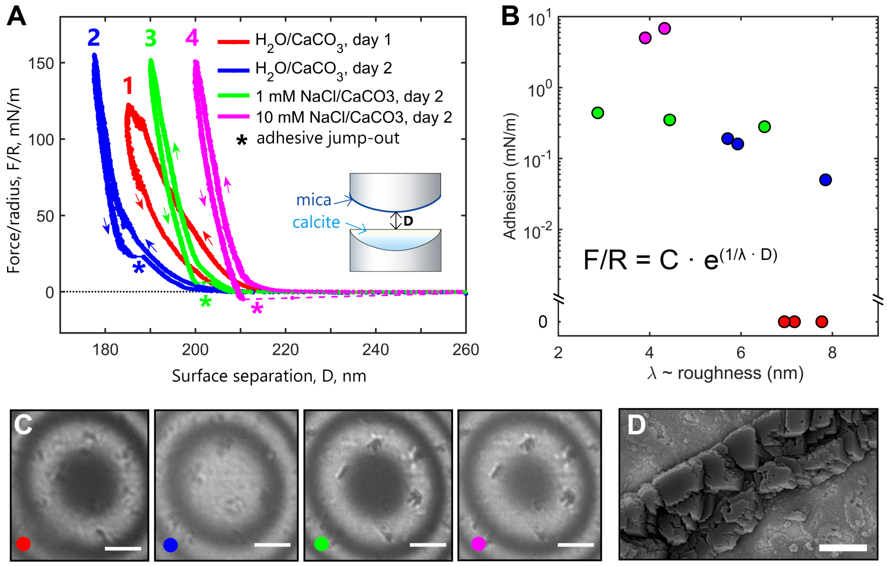

Figure S6: SFA measurements of forces between reactive calcite and inert mica surfaces indicating a progressive increase in the local contact area. **A:** Representative force-separation distance curves measured between rough calcite and smooth mica surfaces in water and NaCl solutions saturated with respect to calcite. The numbers mark the order of measurements. Upon recrystallization, the calcite surfaces progressively grow as shown by the increase in the minimum surface separation (apart from the initial dissolution between solutions 1 and 2). Despite the growth, the local contact area becomes smoother as indicated by the increasing adhesion and decreasing exponential decay length of the force curves measured on approach (see panel B). **B:** Exponential decay length (λ) of the SFA force-distance curves measured on approach (λ is proportional to the local contact roughness) as a function of adhesion between mica and calcite surfaces (for color-coding see the legend in A). **C:** In-situ optical view from the SFA on the contact topography showing the growth of larger calcite asperities in the contact region between two surfaces (outlined by visible Newton rings). The scale bars are 50 μm. **D:** Scanning electron microscopy (SEM) image of one of the larger calcite asperities, showing flat regions on the recrystallized calcite surface. The scale bar is 1 μm.

We observed an analogous process, in which the reactive calcite surface grows locally in confinement to increase the adhesive surface area with the opposing solid surface, in the Surface Forces Apparatus (SFA). We used one rough and reactive calcite surface against a smooth and inert muscovite mica surface (see Figure S6). The forces measured in the

SFA allowed monitoring of the changing contact roughness and surface adhesion during the reactive surface growth.

The preparation of surfaces and details of the SFA experiments have been previously described in Dziadkowiec et al.[10] We used polycrystalline calcite surfaces grown by atomic layer deposition (ALD). Despite using calcite-saturated solutions at all times, we observed recrystallization of calcite in contact with aqueous solutions, which was driven by the disequilibrium morphology of the ALD-deposited calcite crystals.[11] The SFA measurements were performed in micron-scale confinement: the distance between the surfaces was at all times < 1 μm in the most confined region (surfaces were placed in a crossed-cylindrical geometry, yielding a spherical confined contact area with a diameter of $\sim$150 μm).

Figure S6**A** shows a sequence of force-distance curves measured between mica and calcite surfaces in the same contact region. After initial dissolution (solutions 1 to 2), we observed a progressive growth of calcite surfaces (reflected by the increasing minimum surface separation) associated with the decrease in the repulsive force components (indicated by the decreasing exponential decay length of the force curves; Figure S6**B**) and the increasing adhesion.

The forces measured in the SFA were dominated by the repulsive roughness contribution.[3] Thus, the major changes in the repulsive decay length (λ) of the force curves can be almost entirely attributed to the changing 'local' roughness of the calcite surface asperities[11] and not to the slight variations in solution chemistry. According to Benz et al,[12] the local contact roughness (σ) is proportional to the exponential decay length (λ) of the force (F)-distance (D) curves measured on approach according to: $F/R = C * e^{(-D/\lambda)}$, where $\lambda = 2 * \sigma$, and C is a fitting constant. Although, based on the optical in-situ images of the contact region (Figure S6**C**), we can directly observe that the overall roughness of the calcite surfaces was increasing (see dark large calcite asperities appearing with time), the local roughness of the calcite asperities in contact with mica decreased. This smoothing of the contacts led to the increase in an adhesive contact area, as evidenced by the decreasing λ and the increasing

adhesion. The smooth faces on the recrystallized calcite asperities could be evidenced ex-situ with Scanning Electron Microscopy (SEM; Figure S6**D**). As such, calcite growing in the confined SFA geometry did not exert pressure on the opposing mica surface (which would be evidenced by the increase in repulsive force contribution during the force measurements). Instead, the calcite crystals grew locally to maximize the adhesive contact area with mica.

References

(1) Li, L.; Kohler, F.; Røyne, A.; Dysthe, D. Growth of Calcite in Confinement. *Crystals* **2017**, *7*, 361.

(2) Israelachvili, J. N. *Intermolecular and surface forces*; Academic Press, 2011; p 674.

(3) Parsons, D. F.; Walsh, R. B.; Craig, V. S. Surface forces: Surface roughness in theory and experiment. *Journal of Chemical Physics* **2014**, *140*.

(4) Brekke-Svaland, G.; Bresme, F. Interactions between Hydrated Calcium Carbonate Surfaces at Nanoconfinement Conditions. *Journal of Physical Chemistry C* **2018**, *122*, 7321–7330.

(5) Diao, Y.; Espinosa-Marzal, R. M. Molecular insight into the nanoconfined calcite–solution interface. *Proceedings of the National Academy of Sciences* **2016**, *113*, 12047–12052.

(6) Dysthe, D. K.; Renard, F.; Porcheron, F.; Rousseau, B. Fluid in mineral interfaces—molecular simulations of structure and diffusion. *Geophysical Research Letters* **2002**, *29*, 13–14.

(7) Mutisya, S. M.; Kirch, A.; De Almeida, J. M.; Sánchez, V. M.; Miranda, C. R. Molecular Dynamics Simulations of Water Confined in Calcite Slit Pores: An NMR Spin

Relaxation and Hydrogen Bond Analysis. *Journal of Physical Chemistry C* **2017**, *121*, 6674–6684.

(8) Collin, M.; Gin, S.; Dazas, B.; Mahadevan, T.; Du, J.; Bourg, I. C. Molecular Dynamics Simulations of Water Structure and Diffusion in a 1 nm Diameter Silica Nanopore as a Function of Surface Charge and Alkali Metal Counterion Identity. *Journal of Physical Chemistry C* **2018**, *122*, 17764–17776.

(9) Høgberget, J.; Røyne, A.; Dysthe, D. K.; Jettestuen, E. Microscopic modeling of contact formation between confined surfaces in solution. *arXiv:cond-mat* **2020**, *2006.02129*, 1–16.

(10) Dziadkowiec, J.; Javadi, S.; Bratvold, J.; Nilsen, O.; Røyne, A. Surface Forces Apparatus Measurements of Interactions between Rough and Reactive Calcite Surfaces. *Langmuir* **2018**, *34*.

(11) Dziadkowiec, J.; Zareeipolgardani, B.; Dysthe, D. K.; Røyne, A. Nucleation in confinement generates long-range repulsion between rough calcite surfaces. *Scientific Reports* **2019**, *9*, 1–15.

(12) Benz, M.; Rosenberg, K. J.; Kramer, E. J.; Israelachvili, J. N. The deformation and adhesion of randomly rough and patterned surfaces. *The Journal of Physical Chemistry B* **2006**, *110*, 11884–11893.
